# Potential ferroptosis key genes in calcific aortic valve disease

**DOI:** 10.3389/fcvm.2022.916841

**Published:** 2022-08-08

**Authors:** Xiong-Zhi Li, Zhuo-Chao Xiong, Shao-Ling Zhang, Qing-Yun Hao, Ming Gao, Jing-Feng Wang, Jing-Wei Gao, Pin-Ming Liu

**Affiliations:** ^1^Department of Cardiology, Guangzhou Key Laboratory on the Molecular Mechanisms of Major Cardiovascular Disease, Guangdong Provincial Key Laboratory of Arrhythmia and Electrophysiology, Sun Yat-Sen Memorial Hospital, Sun Yat-Sen University, Guangzhou, China; ^2^Department of Endocrinology, Sun Yat-Sen Memorial Hospital, Sun Yat-Sen University, Guangzhou, China; ^3^Department of Radiology, Sun Yat-Sen Memorial Hospital, Sun Yat-Sen University, Guangzhou, China

**Keywords:** calcific aortic valve disease, ferroptosis, biomarkers, non-alcoholic fatty liver disease, hypoxia-inducible factor 1 (HIF-1) signaling

## Abstract

Calcific aortic valve disease (CAVD) is a highly prevalent condition that comprises a disease continuum, ranging from microscopic changes to profound fibro-calcific leaflet remodeling, culminating in aortic stenosis, heart failure, and ultimately premature death. Ferroptosis has been hypothesized to contribute to the pathogenesis of CAVD. We aimed to study the association between ferroptosis genes and CAVD and reveal the potential roles of ferroptosis in CAVD. CAVD-related differentially expressed genes (DEGs) were identified *via* bioinformatic analysis of Datasets GSE51472 and GSE12644 obtained from Gene Expression Omnibus. A ferroptosis dataset containing 259 genes was obtained from the Ferroptosis Database. We then intersected with CAVD-related DEGs to identify the ferroptosis DEGs. Subsequently, protein–protein interaction networks and functional enrichment analyses were performed for ferroptosis DEGs. Then, we used miRWalk3.0 to predict the target pivotal microRNAs. An *in vitro* model of CAVD was constructed using human aortic valve interstitial cells. The qRT-PCR and western blotting methods were used to validate the ferroptosis DEGs identified by the microarray data. A total of 21 ferroptosis DEGs in CAVD containing 12 upregulated and nine downregulated genes were identified. The results of the Gene Set Enrichment Analysis (GSEA) and analysis of the KEGG pathway by WebGestalt indicated that the ferroptosis DEGs were enriched in six signaling pathways among which NAFLD (including IL-6, BID, and PRKAA2 genes) and HIF-1 (including IL-6, HIF-1, and HMOX1 genes) signaling pathways were also verified by DAVID and/or Metascape. Finally, the *in vitro* results showed that the mRNA and protein expression levels of IL-6, HIF-1α, HMOX1, and BID were higher, while the levels of PRKAA2 were lower in the Pi-treated group than those in the control group. However, the addition of ferrostatin-1 (a selective ferroptosis inhibitor) significantly reversed the above changes. Therefore, IL-6, HIF-1α, HMOX1, BID, and PRKAA2 are potential key genes closely associated with ferroptosis in CAVD. Further work is required to explore the underlying ferroptosis-related molecular mechanisms and provide possible therapeutic targets for CAVD.

## Introduction

Calcific aortic valve disease (CAVD) is a common disorder in the elderly, estimated to affect more than 25% of individuals over age 65 years and half of those over age 85 years (1). It is characterized by calcium deposits on the arterial aspect of the aortic valve, which is associated with progression to clinical aortic stenosis and with increased cardiovascular morbidity and mortality (2). Currently, there are no preventive therapies capable of altering its course. With the rapid growth of the aging population worldwide, CAVD is becoming an increasingly prevalent cardiovascular disease. In response to different stimuli, such as endothelial injury, inflammatory reactions, and oxidative stress (3–5), valve interstitial cells (VICs) may undergo a profibrotic and osteoblast-like transformation, which implicates the pathology of CAVD. Recently, it has been described that iron deposits originating from endothelial microfissuring and penetration of senescent erythrocytes into the leaflet fibrosa comprise a central component not only of the progression but also of the initiation of CAVD (6, 7). A novel form of regulated cell death termed ferroptosis, characterized by the iron-dependent intracellular accumulation of lipid peroxidation to lethal levels, is also demonstrated to dysregulate cellular tissue homeostasis (8). However, it is largely unclear whether ferroptosis is involved in the ectopic mineralization of the aortic valve.

Therefore, in this study, we used data mining analytic techniques to screen differentially expressed genes (DEGs) in calcified and normal aortic valve samples. These DEGs were then intersected with the ferroptosis dataset to obtain ferroptosis DEGs. Moreover, to identify crucial biomarkers and establish the pathogenesis of CAVD at the molecular level, we investigated key ferroptosis-related genes that may play essential roles in CAVD. We also constructed *in vitro* experiments by using human aortic VICs to verify the above-mentioned hypothesis. Our results will help to understand ferroptosis in the pathogenesis and highlight potential therapeutic targets for CAVD.

## Materials and methods

### Microarray data extraction and processing

Two original microarray datasets GSE51472 and GSE12644 were obtained from the Gene Expression Omnibus database (GEO), which were detected on the GPL570 platform and founded on the Affymetrix Human Genome U133 Plus 2.0 array. Both were genetic expression profiles of aortic valves from patients with CAVD and healthy controls. GSE51472 contained 15 Finnish patients with CAVD in different stages from normal (*n* = 5), sclerosis (*n* = 5), to calcification (*n* = 5). GSE12644 contained 10 normal and 10 calcified stenotic aortic valves from Canadian patients. To make the median of signal expression intensity of each sample approximately on the same horizontal line after merging and batch calibration, 5 sclerotic valve samples from GSE51472 and the second half of valve samples (Ctrl GSM317368-72 and Case GSM317373-77) from GSE12644 were removed, respectively. The remaining valve samples of both datasets (Supplementary Figures 1A,B) were used for further analysis.

### Differential expression analysis

Principal component analysis (PCA) was performed for an initial review of the dataset and to detect outliers affecting the analysis before DEGs analysis. Uniform manifold approximation and projection (UMAP) was run with the R package *umap* (v0.2.3.1). DEGs between calcified and normal aortic valve samples were identified through the R software (v3.6.3) with the threshold of log_2_|FC| > 0.585 (i.e., the absolute value of fold change >1.5) and adjusted *p*-value <0.05. A *t*-test was used to determine the adjusted *p*-value in the DEG analysis. A ferroptosis dataset containing 259 genes was obtained from the Ferroptosis Database (FerrDb). Then we intersected with DEGs to identify the ferroptosis DEGs. Venny2.1, an online tool, was used to draw a Venn diagram. The heat maps of DEGs were visualized by using the ggplot2 R package (v3.3.3).

### Functional enrichment analysis

DAVID v6.8, Metascape, and WebGestalt were used to perform the functional enrichment analysis, as the 3 different enrichment analysis tools have different algorithms, which can verify the results with each other. Gene set enrichment analysis (GSEA) is a method to classify genes according to the degree of differential expression of two samples (9) and to compare the whole genome expression profiles with predicated gene sets (10). Gene sets with <3 or more than 2,000 genes were filtered out by default in the GSEA of WebGestalt. The Kyoto encyclopedia of genes and genomes (KEGG) analysis was based on the GSEA of WebGestalt. The KEGG pathway and enrichment analysis were also conducted by using DAVID v6.8 to find the pathway in which the target gene was involved. The biological processes of ferroptosis DEGs were annotated by Metascape and the biological pathways of microRNAs (miRNAs) were analyzed using Funrich.

### Protein–protein interaction network and module analysis

STRING was utilized in the protein–protein interaction (PPI) network analysis to retrieve the interaction of target proteins (11). Moreover, the PPI network was visualized by Cytoscape software (v3.9.0) with interaction scores >0.4. The nodes represented genes and the edges represented the links between the genes. Clustering analysis of gene networks was performed using molecular complex detection (MCODE) to determine key PPI network modules (12) (i.e., the key sub-networks), where 1 module point to only 1 function; *p* < 0.05 was considered significant.

### Gene–miRNA interaction networks

Target pivotal miRNAs were predicted by utilizing miRWalk (v3.0), and the key gene–miRNA interaction networks were built by using Cytoscape (13). The predicted results obtained from the miRTarBase and miRWalk database were intersected to improve the accuracy and reliability. The results were filtered with the criteria: *p* < 0.05, seed sequence lengths >7, and 3′-UTR as the target genes-binding regions. We also screened miRNAs that targeted more than two genes.

### Cell culture and cell culture reagents

Human VICs were seeded in 6-well plates and incubated for 7 days in fibroblast medium-2 (containing 5.0% FBS, 1.0% penicillin-streptomycin, 1.0% FGS-2, Cat No. 2331, ScienCell™; Ctrl group), or osteogenic medium with addition of 2.6 mM Na_2_HPO_4_ (Pi group), 10^−7^ M insulin, and 50 μg/ml ascorbic acid (14), or osteogenic medium with additional 10 μM ferrostatin-1 (Fer-1, a selective ferroptosis inhibitor, A4371, APExBIO) (Pi+Fer-1 group) (15). The medium was changed 24 h later and three times per week thereafter. VICs in passages 3–8 were used for the experiments.

### Alizarin red staining

The cells were stained with 1.0% Alizarin red solution (G1038, Service). The cells in six-well plates were washed three times with phosphate-buffered saline (PBS) 1× after indicated treatments, fixed in 4.0% paraformaldehyde (YJ0002, Yongjin) for 30 min, and then washed three times with PBS. About 1.0% of Alizarin red solution was added to the cells for 3–5 min and then washed three times with PBS.

### Western blotting

The cells were lysed with RIPA lysis buffer (P0013B, Beyotime) on ice for 30 min. The supernatants of cells were harvested after centrifugation at 12,000 g for 10 min at 4°C. A BCA protein assay kit (P0012S, Beyotime) was used to determine the total protein concentration. Protein extracts were mixed with loading buffer and boiled at 95°C for 10 min. The boiled samples were loaded onto SDS-polyacrylamide gels followed by electrophoresis and transferred onto polyvinylidene difluoride membranes. The membranes were blocked with TBS-tween containing 5% skim milk, inculcated with either RUNX2 (1:1,000 dilution, S12556, Cell Signaling Technology), BMP2 (1:1,000 dilution, AF0075, Beyotime), HMOX-1 (1:1,000 dilution, AF1333, Beyotime), BID (1:1,000 dilution, AF6306, Beyotime), IL-6 (1:1,000 dilution, AF7236, Beyotime), HIF-1α (1:1,000 dilution, CSB-PA002906, CUSABIO), PRKAA2 (1:2,000 dilution, CSB-PA805325LAO1HU, CUSABIO), β-actin (1:5,000 dilution, AF5003, Beyotime), or GAPDH (1:10,000 dilution, S2118, Cell Signaling Technology) antibodies overnight at 4°C. The membranes were washed and inculcated with HRP-labeled secondary antibody (1:5,000 dilution, ab205718, Abcam). Detection was done using clarity western ECL (WBKLS0100, Millipore). The images were acquired through the e-Blot Touch Imager and semiquantitative analyses were performed by the FIJI software.

### Quantitative real-time PCR

The quantitative real-time PCR (qRT-PCR) was used to validate the significantly regulated mRNAs of ferroptosis DEGs identified by the microarray results. Total RNA was extracted from human VICs using TRIzol reagent (Cat No. 15596018, Invitrogen). The quality of total RNA was monitored by NanoDrop One/OneC (Thermo Scientific, United States). One microgram of RNA was reverse transcribed into cDNA using PrimeScript™ RT Master Mix kit (RR036, Takara). The cDNA was used as a template for qRT-PCR. qRT-PCR was performed using a TB Green Premix Ex Taq qPCR kit (RR420, Takara) on a ROCHE LightCycler 480 Real-Time System in a total volume of 10 μl with conditions of 95°C denaturation for 30 s followed by 40 cycles of 95°C for 5 s and 60°C for 30 s. Supplementary Table 1 shows the sequences of the specific primers designed custom by IGE Biotechnology (Guangzhou, China).

### Statistical analysis

Statistical analysis was performed using IBM SPSS v22.0 (SPSS Inc.) and graphs were drawn with GraphPad Prism v8.0.1 (GraphPad Software, Inc.). All values were shown as mean ± standard deviation (SD) of ≥3 independent experiments. Student's *t*-test was used in DEGs analysis to compare the difference between the two groups. One-way analysis of variance followed by the Bonferroni test were used for comparison of >2 groups. Differences at the *p*-value or adjusted *p*-value (by FDR) <0.05 level were considered statistically significant.

## Results

### Identification of ferroptosis DEGs

**Figure 1:**
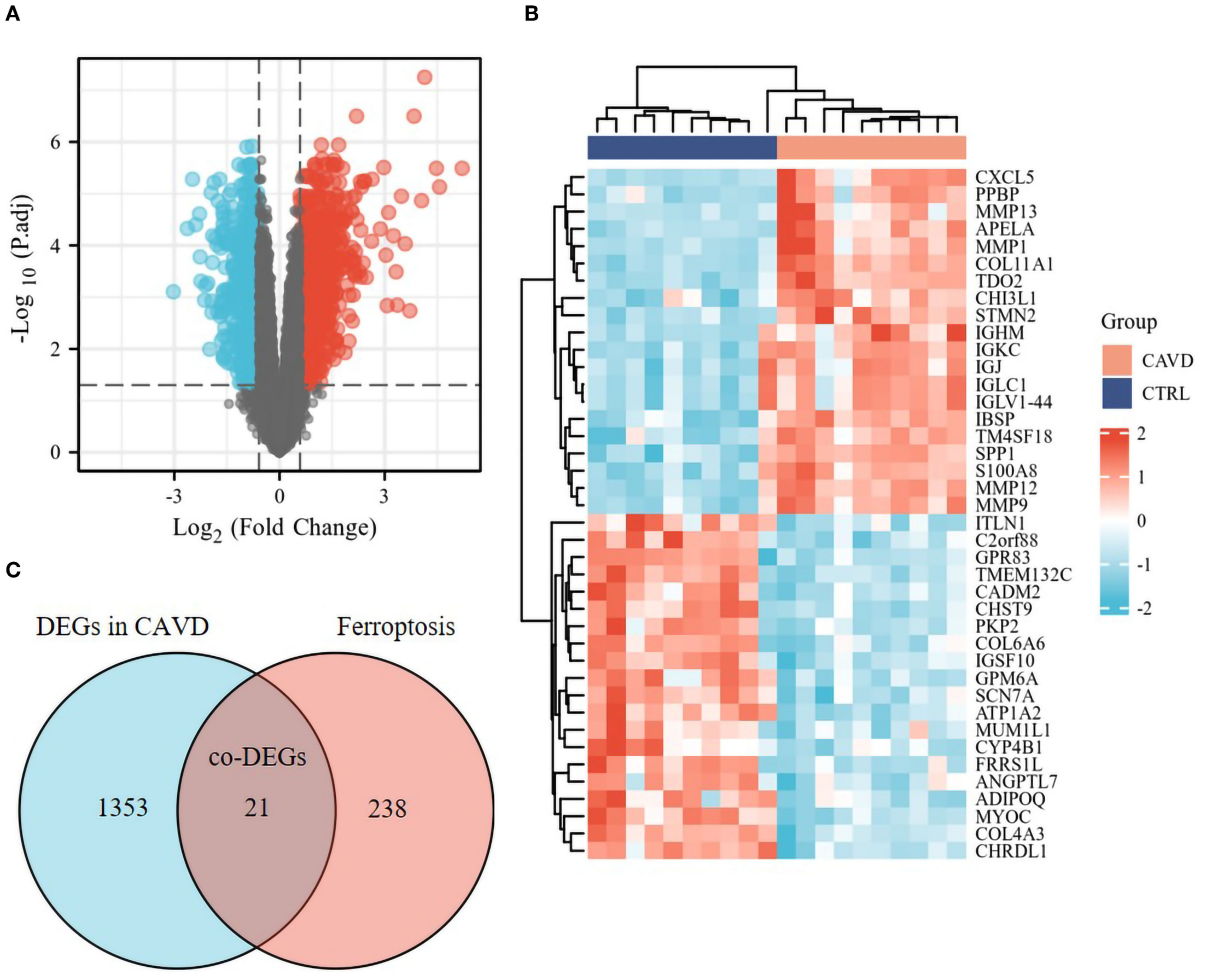
Identification of the ferroptosis DEGs in CAVD. **(A)** Volcano plot of DEGs in CAVD. Volcano plots were drawn after the combination of GSE12644 and GSE51472 gene chips. 21,655 genes remained after removing the null ID value and 1,374 DEGs, including 795 upregulated (red dots) and 579 downregulated genes (blue dots), met the threshold set as fold change >1.5 and adjusted *p*-value <0.05 in CAVD. **(B)** Heatmap of DEGs in CAVD. Heatmap shown the upregulated (red, positive value) and downregulated (blue, negative value) top 20 DEGs in these samples. **(C)** Venn diagrams of the ferroptosis DEGs. Venn diagram indicated 21 co-DEGs (i.e., ferroptosis DEGs) from the intersection of the ferroptosis dataset (259 genes) with DEGs in CAVD above.

The median of each sample was basically on a horizontal line after batch correction, which indicated good normalization among samples (Supplementary Figures 1A,B). The changes in PCA (Supplementary Figures 1C,D) and UMAP (Supplementary Figures 1E,F) charts after batch correction revealed that the samples of each group are separated, which suggested significant differences likely between the two groups. As depicted in the flowchart (Supplementary Figure 2), DEGs set in CAVD were generated by comparing the expression profiles between calcified aortic valves and healthy aortic valves from GSE51472 and GSE12644. The Volcano plots and heatmaps of DEGs in CAVD are shown in Figures 1A,B. Subsequently, the DEGs set was intersected with the relevant ferroptosis gene set containing 259 genes from the FerrDb to obtain the co-DEGs, termed ferroptosis DEGs (Figure 1C), which contained 12 upregulated and nine downregulated genes (Table 1). Ferroptosis DEGs were sorted as ferroptosis marker, ferroptosis driver, and ferroptosis suppressor through the FerrDb online tool (Table 2).

**Table 1:** Ferroptosis DEGs including 12 upregulated and 9 downregulated genes in CAVD.

**Gene symbol**	**Adj. *p*-value**	**Fold change**	**Gene title**	**Gene ID**
HMOX1	0.00027724	3.48685	Heme oxygenase 1	203665_at
RRM2	0.00492335	2.291775	Ribonucleotide reductase regulatory subunit M2	209773_s_at
SLC2A3	0.00689246	2.262022	Solute carrier family 2 member 3	202499_s_at
DPP4	0.0003661	2.190238	Dipeptidyl peptidase 4	203717_at
IL-6	0.01116927	2.039615	Interleukin 6	205207_at
ENPP2	0.00017783	1.880837	Ectonucleotide pyrophosphatase/phosphodiesterase 2	209392_at
ALOX5	0.00050149	1.862017	Arachidonate 5-lipoxygenase	204446_s_at
CAPG	7.45E-05	1.852201	Capping actin protein, gelsolin like	201850_at
BID	7.46E-05	1.668894	BH3 interacting domain death agonist	227143_s_at
CYBB	0.00366743	1.663937	Cytochrome *b*-245 beta chain	203923_s_at
NCF2	0.00574006	1.642676	Neutrophil cytosolic factor 2	209949_at
HIF-1α	5.42E-05	1.575808	Hypoxia-inducible factor 1 subunit alpha	200989_at
NQO1	0.00011217	−1.52207	NAD(P)H quinone dehydrogenase 1	201468_s_at
AKR1C1	0.00734824	−1.52269	Aldo-keto reductase family 1 member C1	204151_x_at
PSAT1	0.04036023	−1.59355	Phosphoserine aminotransferase 1	223062_s_at
PLIN4	0.01656886	−1.60675	Perilipin 4	228409_at
CDO1	0.00534642	−1.65061	Cysteine dioxygenase type 1	204154_at
VLDLR	0.00024013	−1.86915	Very low density lipoprotein receptor	209822_s_at
PRKAA2	0.00640449	−1.90566	Protein kinase AMP-activated catalytic subunit alpha 2	227892_at
TF	0.00071021	−2.83968	Transferrin	203400_s_at
ANGPTL7	0.00192741	−3.39843	Angiopoietin like 7	206423_at

**Table 2:** Classification of the ferroptosis DEGs.

**Marker (*n* = 12)**	**Driver (*n* = 9)**	**Suppressor (*n* = 5)**	
HMOX1, RRM2, SLC2A3, IL-6, ALOX5, CAPG, NCF2, PSAT1, PLIN4, VLDLR, TF, ANGPTL7	HMOX1, DPP4, ALOX5, BID, CYBB, HIF-1α, CDO1, PRKAA2, TF	HMOX1, ENPP2, HIF-1α, NQO1, AKR1C1	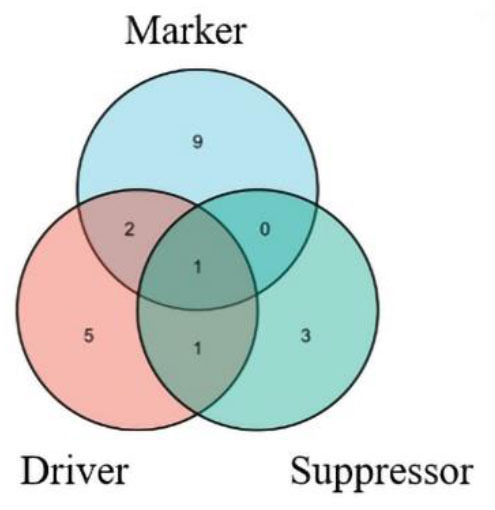

Marker (n = 12) + Driver (n = 9) + Suppressor (n = 5) = 26, which was larger than the ferroptosis DEGs count (21), because of 4 multi-annotated genes (underlined) as shown in the Venn diagram.

### Enrichment pathway and analysis of ferroptosis DEGs

**Figure 2:**
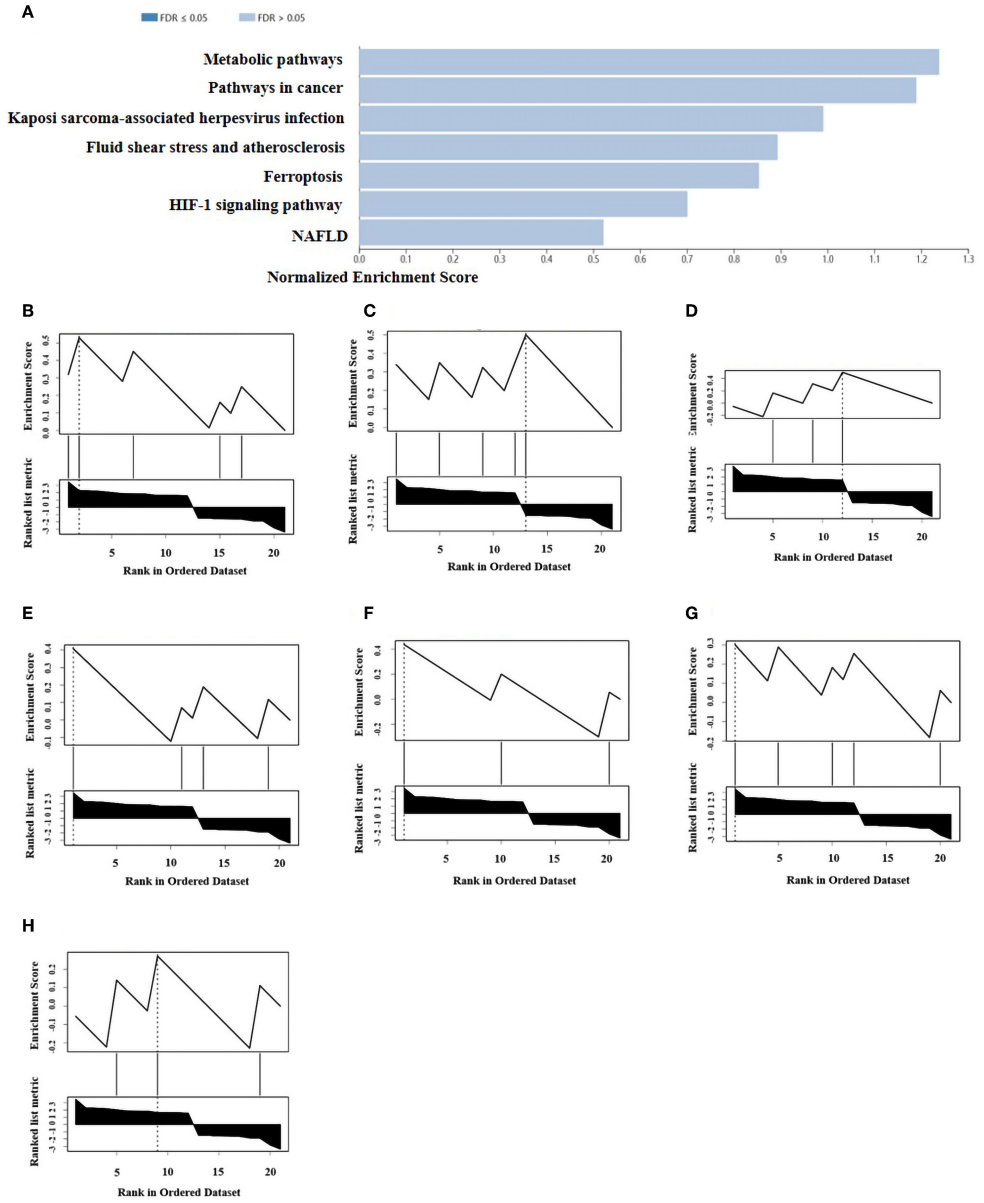
GSEA and KEGG pathway analysis of the ferroptosis DEGs. **(A)** Pathways enriched of the ferroptosis DEGs by GSEA and KEGG analysis. Enrichment results (weighted set cover) indicated the genes significantly enriched were linked to several signaling pathways. **(B)** Enrichment plot: metabolic pathway. **(C)** Enrichment plot: pathways in cancer. **(D)** Enrichment plot: Kaposi sarcoma-associated herpesvirus infection. **(E)** Enrichment plot: fluid shear stress and atherosclerosis. **(F)** Enrichment plot: ferroptosis. **(G)** Enrichment plot: HIF-1 signaling pathway. **(H)** Enrichment plot: NAFLD. WebGestalt was used in GSEA of ferroptosis DEGs for visualization, which filtered the genes according to the number of genes contained in the gene set, with the ranges of gene number from 3 to 2,000.

**Figure 3:**
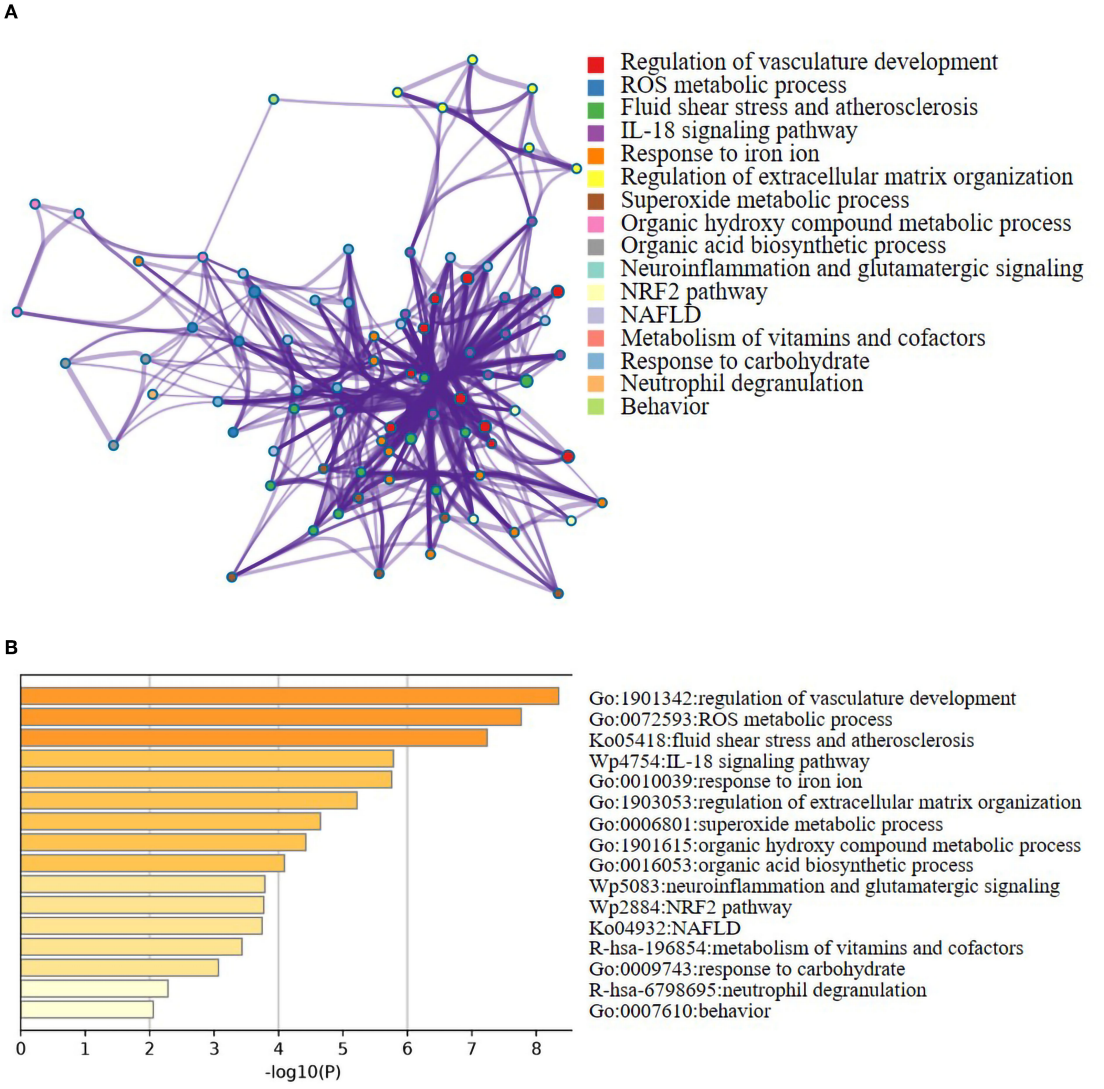
Networks and the bar chart of enriched terms. **(A)** Networks of enriched terms of the 21 ferroptosis DEGs analyzed by Metascape. **(B)** The bar chart of 16 biological pathways based on the *p*-value (<0.01) and the percentage of the gene number.

A total of 21 ferroptosis DEGs in CAVD were uploaded to the WebGestalt. GSEA and analysis of the KEGG pathway were performed. The result of the ferroptosis DEGs indicated that genes significantly enriched were linked to metabolic pathways, pathways in cancer, Kaposi sarcoma-associated herpesvirus infection, fluid shear stress and atherosclerosis, ferroptosis, hypoxia-inducible factor 1 (HIF-1) signaling pathway, and non-alcoholic fatty liver disease (NAFLD; Figures 2A–H). Next, the ferroptosis DEGs were also uploaded to DAVID and Metascape to analyze the biological pathway and processes. KEGG functional analysis obtained from DAVID indicated that biological processes were remarkably enriched in the HIF-1 signaling pathway, NAFLD, and mineral absorption (Table 3). Finally, the results obtained from Metascape revealed that biological processes containing regulation of vasculature development, ROS metabolic process, and response to iron ion were significantly activated in the gene sets. The significantly activated biological pathways were NAFLD, fluid shear stress and atherosclerosis, and interleukin-18 signaling pathway (Figure 3). Notably, NAFLD [containing interleukin-6 (IL-6), BH3 interacting domain death agonist (BID), and protein kinase AMP-activated catalytic subunit alpha 2 (PRKAA2) genes] was the major biological pathway involved and was consistently identified by WebGestalt, DAVID, and Metascape. Similarly, the HIF-1 signaling pathway [containing IL-6, heme oxygenase-1 (HMOX1), and HIF-1α genes] was also verified by both WebGestalt and DAVID.

**Table 3:** Three biological pathways were significantly activated by using DAVID.

**Term**	**Genes**	**Count**	**%**	***p*-Value**	**Benjamini**
HIF-1 signaling pathway	IL-6, HMOX1, HIF-1α, TF	4	19	1.10E-03	8.50E-02
NAFLD	IL-6, BID, PRKAA2	3	14.3	4.20E-02	1.00E+00
Mineral absorption	TF, HMOX1	2	9.5	9.20E-02	1.00E+00

### Protein–protein interaction network analysis of ferroptosis DEGs

**Figure 4:**
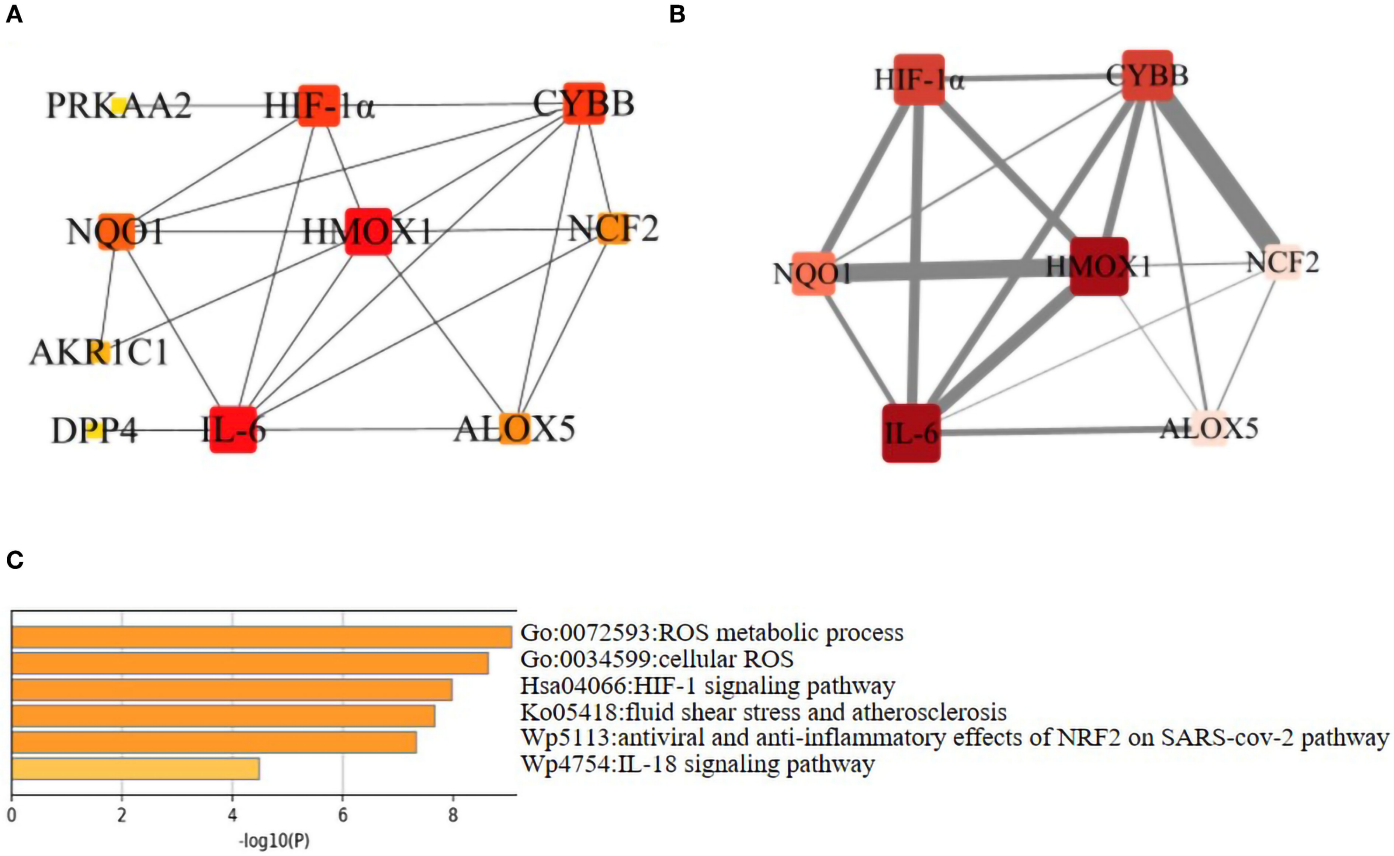
Network analysis of ferroptosis DEGs. **(A)** PPI network of the ferroptosis DEGs. Visualization of PPI networks containing 11 nodes and 22 edges was obtained with interaction scores >0.4. Ten of the 21 genes did not form a molecular network with other molecules (not shown). **(B)** PPI network of the key ferroptosis DEGs. The key module was identified by the Cytokeyba plug-in of Cytoscape software for clustering analysis of the key gene network. The node gene cluster with the highest score constructed by the MCODE plug-in in Cytoscape consists of seven genes. Genes were represented by nodes (the size of nodes referred to the degree, the color depth of nodes referred to the neighborhood connectivity, i.e., closeness), and links between genes were represented by edges (the thickness of gray edges referred to a combined score ranging from 3.2 to 4.0). **(C)** Functional enrichment analysis of seven key ferroptosis DEGs. It was shown that the HIF-1 signaling pathway was significantly enriched. These six biological pathways were drawn based on the *p*-value (<0.0001) and the percentage of the gene number.

The constructed PPI networks, including 11 nodes and 22 edges with an interaction score>0.4, were generated using Cytoscape (v3.9.0; Figure 4A). A total of 10 of the 21 ferroptosis DEGs did not form a molecular network with other molecules (not shown). A key module containing seven key genes screened by MCODE was established (Figure 4B). Genes of interest were selected from the key module involved in the HIF-1 signaling pathway with the highest MCODE scores, including IL-6, HIF-1α, and HMOX1. Moreover, the functional analysis for genes in the key module using Metascape revealed that the seven genes were mainly involved in the HIF-1 signaling pathway, ROS metabolic process, cellular response to oxidative stress, fluid shear stress, and atherosclerosis (Figure 4C).

### Further miRNA interaction and mining

**Figure 5:**
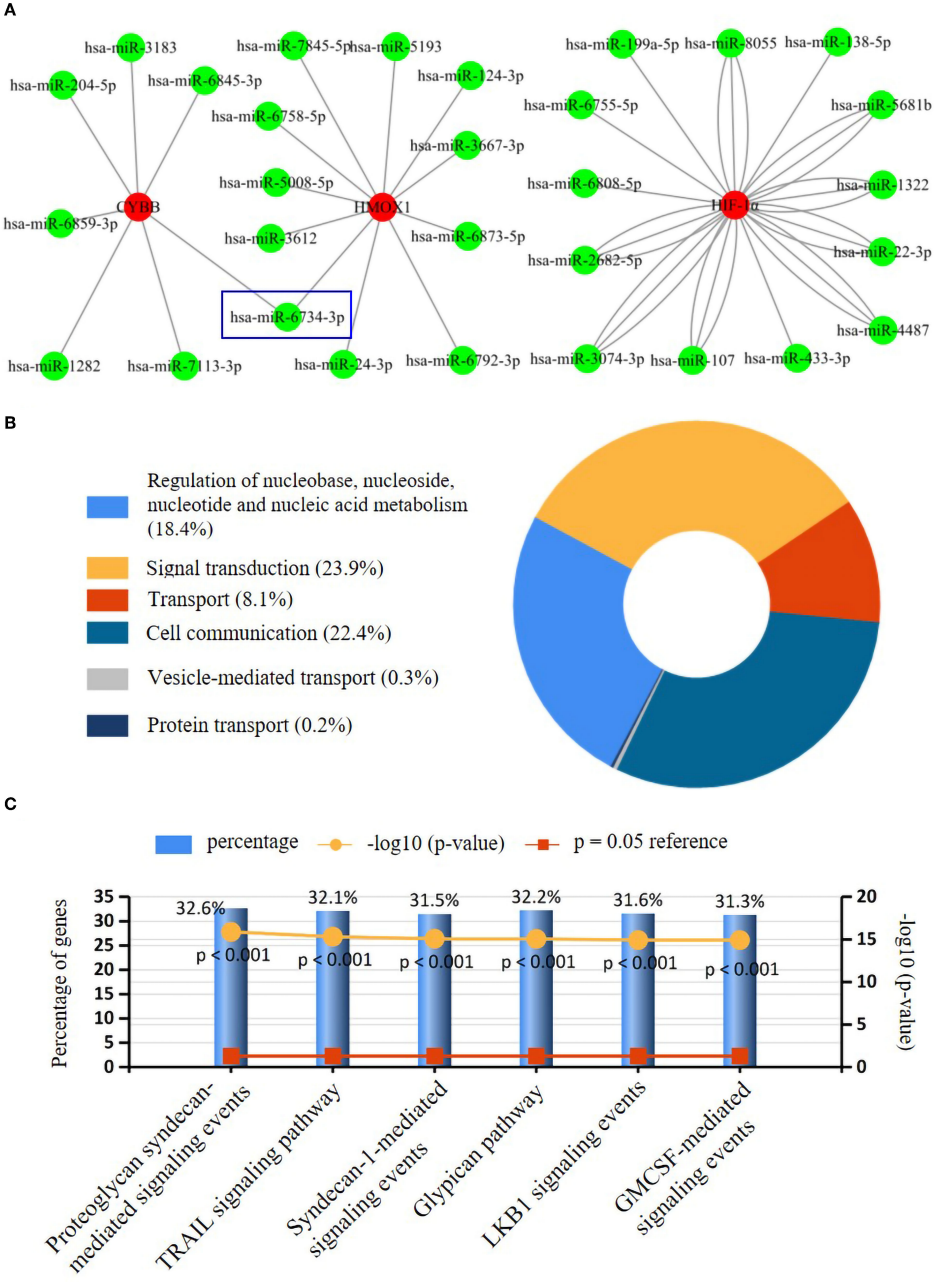
Interaction networks between key ferroptosis DEGs and their targeted miRNAs. **(A)** Interaction network between genes of the key ferroptosis DEGs and its targeted miRNAs. Genes were colored in red, and miRNAs were colored in green. Only has-miR-6743-3p with higher amounts of cross-linked genes (=2, HMOX1, and CYBB) was shown in the blue wireframe. **(B)** The molecular function of key ferroptosis DEGs-related miRNAs. **(C)** The top three biological pathways (Proteoglycan syndecan mediated-signaling events, TRAIL signaling pathway, and glypican pathway) enriched of the key ferroptosis DEGs-related miRNAs.

Seven key ferroptosis DEGs in CAVD were screened and then put into gene–miRNA analysis by using the miRWalk2.0 software. The cross-linked miRNAs were screened by both miRWalk and miRTarBase databases to improve the reliability and accuracy of the results. A total of 30 miRNA expression genes were selected. The miRNA with higher amounts of a cross-linked gene (*n* ≥ 2) was hsa-miR-6734-3p (Figure 5A). The molecular function of key ferroptosis DEGs-related miRNAs analyzed using Funrich software was significantly enriched in the signal transduction (23.9%), cell communication (22.4%), regulation of nucleobase, nucleoside, nucleotide, and nucleic acid metabolism (18.4%) and transport (8.1%; Figure 5B). Proteoglycan syndecan-mediated signaling events, tumor necrosis factor-related apoptosis-inducing ligand (TRAIL) signaling pathway, and glypican pathway were the top 3 biological pathways enriched by these miRNAs (Figure 5C).

### Potential biomarker expression by qRT-PCR and western blotting

**Figure 6:**
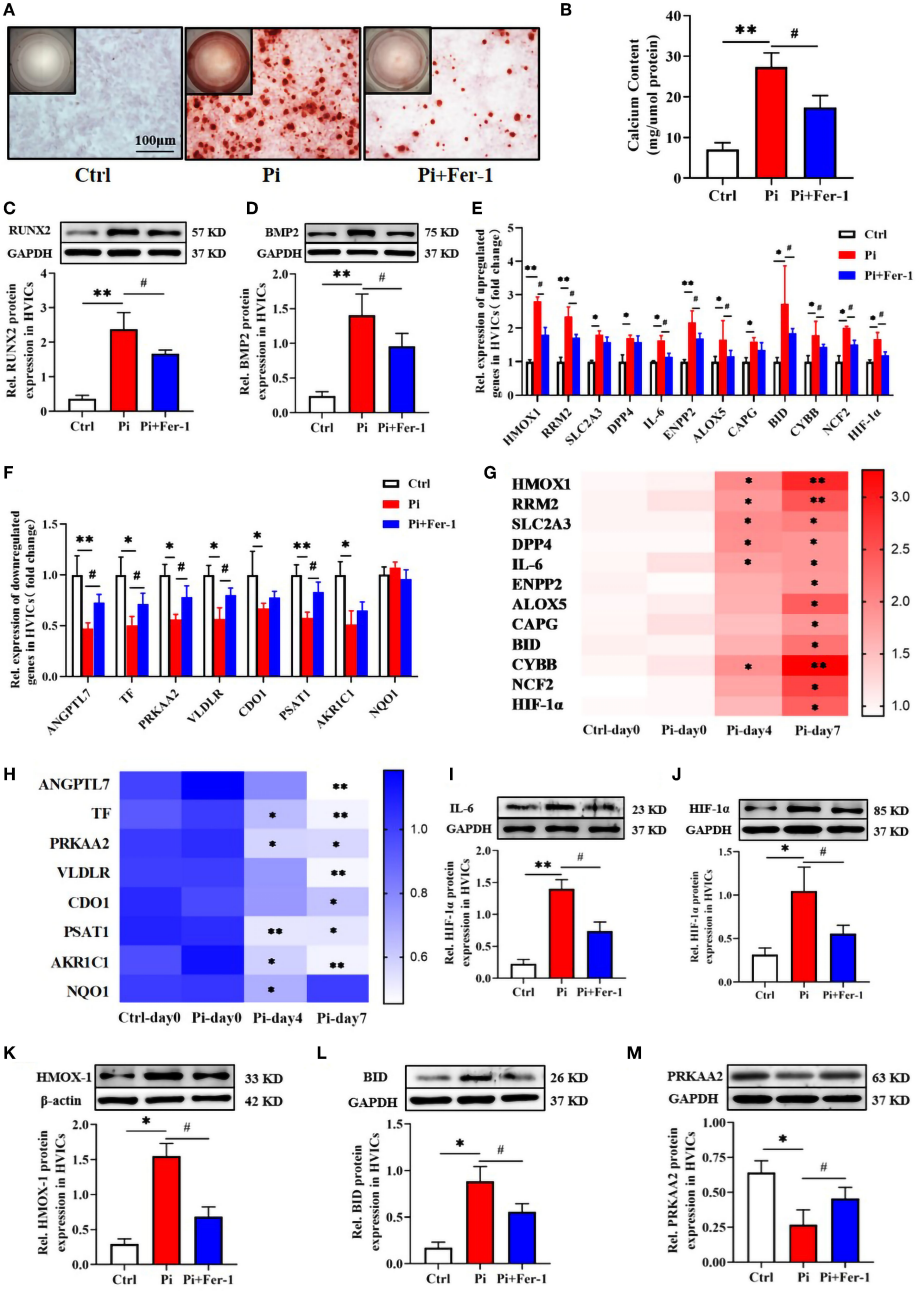
Validation of the potential biomarker expression *in vitro*. **(A)** Calcium deposition in VICs visualized using Alizarin red stain (red signal). Representative macroscopic (top left corner) and microscopic (the rest, scale bar: 100 μm) findings were shown. **(B)** Quantification of calcium deposition in VICs normalized to the protein content. **(C,D)** Representative bands of Western blotting and semiquantitative analysis of RUNX2, BMP2 protein in VICs. **(E,F)** Transcriptional expression of ferroptosis DEGs expressed as fold changes. **(G,H)** Heatmap of ferroptosis DEGs expression over time shown as fold changes. The row label represents gene name, the column label represents subgroup, and the vertical colormap in the right represents fold changes. **(I–M)** Representative bands of Western blotting and semiquantitative analysis of IL-6, HMOX-1, HIF-1α, BID, and PRKAA2 protein in VICs, respectively. *n* = 3–4 per group for *in vitro* experiments. Values shown were mean ± SD. *p < 0.05 vs. Ctrl or Ctrl-day 0; **p < 0.01 vs. Ctrl or Ctrl-day 0, ^#^*p* < 0.05 vs. Pi.

As proven by Alizarin red S staining (Figure 6A), the calcium content assay (Figure 6B), and western blotting (Figures 6C,D), the calcium deposition and osteogenic markers (RUNX2, BMP2) in human VICs of Pi-treated group were significantly higher than in control group, which indicated the osteogenic model of VICs was constructed successfully. However, further addition of Fer-1 significantly inhibited VIC osteogenic differentiation and calcification (Figures 6A–D).

All 21 ferroptosis DEGs were verified *in vitro* experiments. The qRT-PCR results regarding these genes were consistent with the bioinformatics analysis described above except for NAD(P)H quinone dehydrogenase 1 (NQO1). Compared with the control group, 12 genes were upregulated and 8 genes were downregulated in the Pi-treated group, which were at least in part reversed by the addition of Fer-1 (Figures 6E,F). Meanwhile, the transcriptional change in ferroptosis DEGs expression over time was in a time-dependent manner except for NQO1 expression (Figures 6G,H). Importantly, we focused on genes involved in NAFLD (including IL-6, BID, and PRKAA2), and HIF-1 (including IL-6, HIF-1, and HMOX1) signaling pathways. Consistently, the protein expression levels of IL-6 (Figure 6I), HIF-1α (Figure 6J), HMOX1 (Figure 6K), and BID (Figure 6L) were higher, while the levels of PRKAA2 (Figure 6M) were lower in the Pi-treated group than those in the control group. However, the addition of Fer-1 significantly reversed the changes of these proteins induced by Pi treatment (Figures 6I–M), providing evidence for the roles of ferroptosis in VIC osteogenic differentiation and calcification through NAFLD and HIF-1 signaling pathways.

## Discussion

This study for the first time identified the key ferroptosis-related genes involved in CAVD. By analysis of microarray datasets and *in vitro* confirmative experiments, we demonstrated that the NAFLD (including IL-6, BID, and PRKAA2 genes) and HIF-1 signaling pathways (including IL-6, HIF-1, and HMOX1 genes) were ferroptosis-related pathways in CAVD. The study provides novel insights into the roles of ferroptosis during the development of CAVD.

CAVD encompasses a disease spectrum ranging from aortic valve sclerosis (i.e., fibro-calcific leaflet remodeling without significant impairment in leaflet motion and aortic orifice narrowing) to severe left ventricular outflow obstruction by calcific aortic valve stenosis (16). CAVD was previously considered a degenerative disease due to the gradual calcium deposition in the aortic valve while getting older. It is now generally accepted that CAVD is a complicated and active process and various physiological and pathological conditions are involved in the regulation, such as lipoprotein deposition, inflammatory response, ROS formation, and VIC apoptosis (17). The loading of valvular disease is anticipated to ascend, in consideration of the prolonged life expectancy and the current insufficiency of effective preventive measures, emphasizing the need to deepen our discernment of the pathophysiology of retrogressive heart valve diseases (18, 19).

Ferroptosis is characterized by the iron-dependent intracellular accumulation of lipid ROS, which are closely involved and eventually result in lipid peroxidation, thereby contributing to cell death and dysregulation of cellular tissue homeostasis (8). ROS-associated cell death and apoptosis are considered classical mechanisms of calcific nodule formation and expansion of amorphous calcium in CAVD (20). Thus, it is suggested that inhibition targeting ferroptosis may be a possible strategy for regression of CAVD. In this study, ferroptosis (Figure 2F), ROS metabolic process (Figures 3A,B), and NAFLD (containing IL-6, BID, PRKAA2) signaling were identified as the activated biological processes or pathways involved in CAVD, and particularly, NAFLD was validated by WebGestalt, DAVID, and Metascape, which was consistent with the previous findings (6, 21). Iron overload was observed in patients with NAFLD and the liver damage could be attenuated by iron removal (22, 23). Therefore, the pathological process of NAFLD is closely associated with ferroptosis due to iron overload and lipid peroxidation (23). In addition, it was shown that iron deposits and penetration of senescent erythrocytes into the lea?et fibrosa comprised a central component not only of the initiation but also of the progression of CAVD (6). Then, the coculture of primary VICs derived from noncalcified valves with senescent erythrocytes resulted in a global inflammatory and osteoblastic phenotype, reflected by an elevated expression of IL-6, BMP2, and the formation of calcium deposits (6). Iron is a vital element for the function of many proteins and enzymes in physiology. It is well established that iron potently catalyzes the generation of toxic ROS and thereby induces oxidative damage of lipids (7). ROS in turn can activate redox-sensitive transcription factors whose targets include proinflammatory cytokines, such as IL-6 in CAVD. It was documented that the expression levels of IL-6 were significantly elevated in both human calcified aortic valves and VICs treated with a calcifying medium (24), which were accompanied by an upregulation of RUNX2 and osteopontin (25). However, the roles of BID and PRKAA2 found in this study during CAVD are not well understood yet. A recent study reported a novel mechanism linking ferroptosis and CAVD development, in which iron promoted Slc7a11-deficient VIC osteogenic differentiation *via* aggravating ferroptosis, and in turn accelerated the progression of CAVD (26). Although the differential expression of Slc7a11 was not detected in our study, we hypothesized that the ferroptosis genes occurred at different stages and coordinately participate in the development of CAVD. Further research is warranted to assess the specific mechanisms for IL-6, especially BID and PRKAA2 in the progression of CAVD.

Hypoxia is one of the stimulatory factors in mid to late valve disease progression and vascular calcification (27). HIF-1, a central oxygen-sensitive transcription factor composed of an oxygen-regulated subunit HIF-1α (or its paralogs HIF-2α and HIF-3α) and a constitutive subunit HIF-1β, was involved in response to hypoxia in CAVD (28, 29). Hypoxia increased the ROS levels in a variety of pathological situations, including CAVD and vascular calcification, which could result in the activation of HIF-1α (27). However, the repression of the HIF-1 signaling pathway in turn caused excessive production of mitochondrial ROS (30). Therefore, this indicates that HIF-1 signaling potentially plays a role in CAVD-related ferroptosis. In this study, HIF-1 signaling was enriched in CAVD by KEGG pathway analysis verified both with WebGestalt and DAVID. In detail, IL-6, HIF-1α, and HMOX1 were included and selected from the key module involved in this pathway with the highest MCODE scores. IL-6 could perturb iron homeostasis and cause chondrocyte ferroptosis. However, pharmacological inhibition of the IL-6 partially abolished the ferroptosis-inducing effects (31). Emerging data indicate that HIF-1 signaling is possibly involved in iron homeostasis. Ni et al. (32) showed that HIF-1α decreased ferritinophagy and autophagy flux of osteoclasts under hypoxia. Additionally, HIF-2α was a crucial component of the signaling mechanism that mediates iron absorption following iron deficiency (33). HMOX1, an inducible isoform of heme oxygenase, can facilitate the degradation of heme into equimolar amounts of free iron, carbon monoxide, and biliverdin (34). Upregulation of HMOX1 inhibited VIC osteogenic differentiation *in vitro* and served as a potential target for CAVD (35). More importantly, HMOX1 knockdown inhibited iron overload and the production of ROS, consequently alleviating lipid peroxidation, which resulted in a reduction in ferroptosis (36). Taken together, these data indicate that differential expression of IL-6, HIF-1α, and HMOX1 may play critical roles in CAVD-related ferroptosis. Notably, our finding showed that ferrostatin-1, a potent and selective ferroptosis inhibitor, significantly reversed the changes in the mRNA and protein levels (IL-6, BID, PRKAA2, HIF-1α, and HMOX1) in the calcifying medium, repressed osteogenic differentiation, and calcification of VICs. Thus, therapies that target ferroptosis have the potential in retarding the development of CAVD.

MicroRNAs are endogenous non-coding RNA molecules that bind to the 3′-UTR ends of mRNAs transcribed from the target genes, which result in degrading or inhibiting the translation of the target genes (37). In this study, we identified 30 miRNAs interacting with key ferroptosis DEGs in the development of CAVD, among which reduction of miR-204-5p and miR-138-5p, and the upregulation of miR-22-3p were previously reported in CAVD and VICs treated with calcifying medium (38–40). Moreover, miR-199a-5p could enhance osteogenesis maturation by inhibiting the HIF1α-Twist1 pathway in mesenchymal stem cells (41), which partially supported our bioinformatics prediction. Our findings suggested that the mRNA-miRNA connections may play a role in CAVD, and further studies are needed to determine the specific molecular mechanism and the effects of those unexplored miRNAs.

Several limitations of this study need to be highlighted. First, a microarray analysis was adopted in the study and all the results were based on gene expression values. As mRNA expression level is not always a good indicator of protein expression level, the biomarkers of this study should be interpreted as genes, not proteins. Second, the sample size of the microarray datasets was relatively small and remained confounding even though we excluded the ferroptosis DEGs in the stage of progressive sclerosis for matching during the integrated analysis. Third, our validation was limited to *in vitro* experiments. Further *in vivo* data and clinical trials are warranted to investigate the role of ferroptosis in the development and progression of CAVD.

## Conclusion

The study reveals that IL-6, HIF-1α, HMOX1, BID, and PRKAA2 are key genes associated with ferroptosis in CAVD. NAFLD and HIF-1 signaling pathways may play crucial roles in CAVD-related ferroptosis. Further studies are required to explore the underlying ferroptosis-related molecular mechanisms for the pathogenesis of CAVD and provide potential therapeutic targets.

## Data availability statement

The datasets presented in this study can be found in online repositories. The names of the repository/repositories and accession number(s) can be found at: https://www.ncbi.nlm.nih.gov/genbank/, GSE51472 and GSE12644.

## Ethics statement

Written informed consent was obtained from the individual(s) for the publication of any potentially identifiable images or data included in this article.

## Author contributions

X-ZL and Z-CX: conceptualization, methodology, validation, investigation, data curation, writing—original draft, and visualization. Q-YH and MG: conceptualization, methodology, validation, and investigation. J-WG: funding acquisition, writing—review, and editing. S-LZ: funding acquisition and supervision. P-ML: funding acquisition, review and editing, and supervision. J-FW: supervision. All authors read and approved the final version of the manuscript.

## Funding

This work was supported by grants from the National Natural Science Foundation of China [82170457, 81900379, 81870315, and 81970683] and the Natural Science Foundation of Guangdong Province [2022A1515011920].

## Conflict of interest

The authors declare that the research was conducted in the absence of any commercial or financial relationships that could be construed as a potential conflict of interest.

## Publisher's note

All claims expressed in this article are solely those of the authors and do not necessarily represent those of their affiliated organizations, or those of the publisher, the editors and the reviewers. Any product that may be evaluated in this article, or claim that may be made by its manufacturer, is not guaranteed or endorsed by the publisher.

## Supplementary material

Supplementary Figure 1 PCA and UMAP analysis before obtaining DEGs. **(A)** The expression signal intensity of each sample before batch correction. **(B)** The expression signal intensity of each sample after batch correction. **(C)** PCA chart before batch correction. **(D)** PCA chart after batch correction. **(E)** UMAP chart before batch correction. **(F)** UMAP chart after batch correction.

Supplementary Figure 2 Flowchart of data analysis.

Supplementary Table 1 Specific primers of the ferroptosis DEGs used for qRT-PCR.
