# Supplementary figures and images for: Potential ferroptosis key genes in calcific aortic valve disease
Source: Front Cardiovasc Med. 2022 Aug 8;9:916841. doi: 10.3389/fcvm.2022.916841 (PMC9395208; doi:10.3389/fcvm.2022.916841)

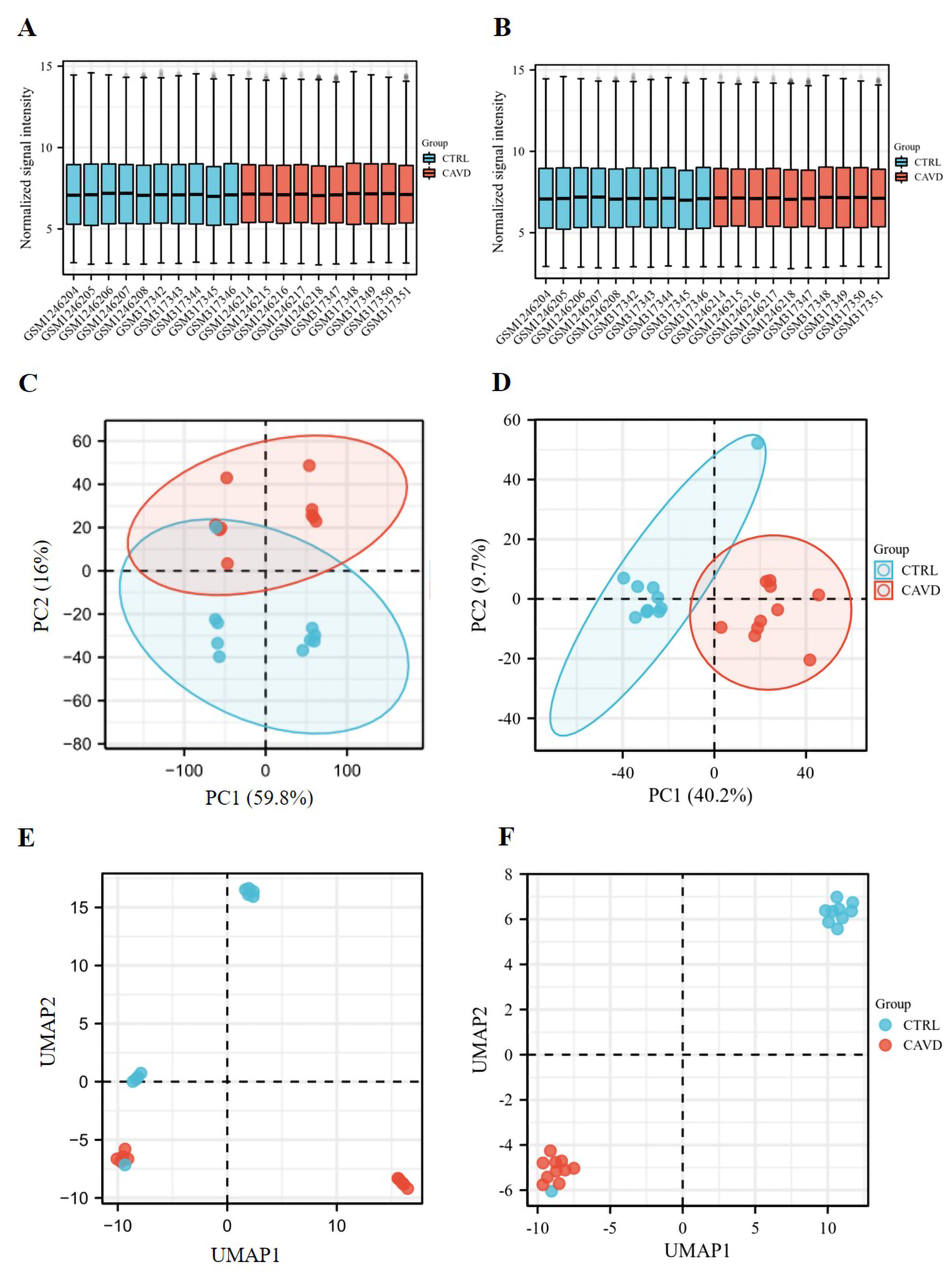

Supplement: Supplementary Figure 1 — PCA and UMAP analysis before obtaining DEGs. (A) The expression signal intensity of each sample before batch correction. (B) The expression signal intensity of each sample after batch correction. (C) PCA chart before batch correction. (D) PCA chart after batch correction. (E) UMAP chart before batch correction. (F) UMAP chart after batch correction. [file Image_1.TIF]

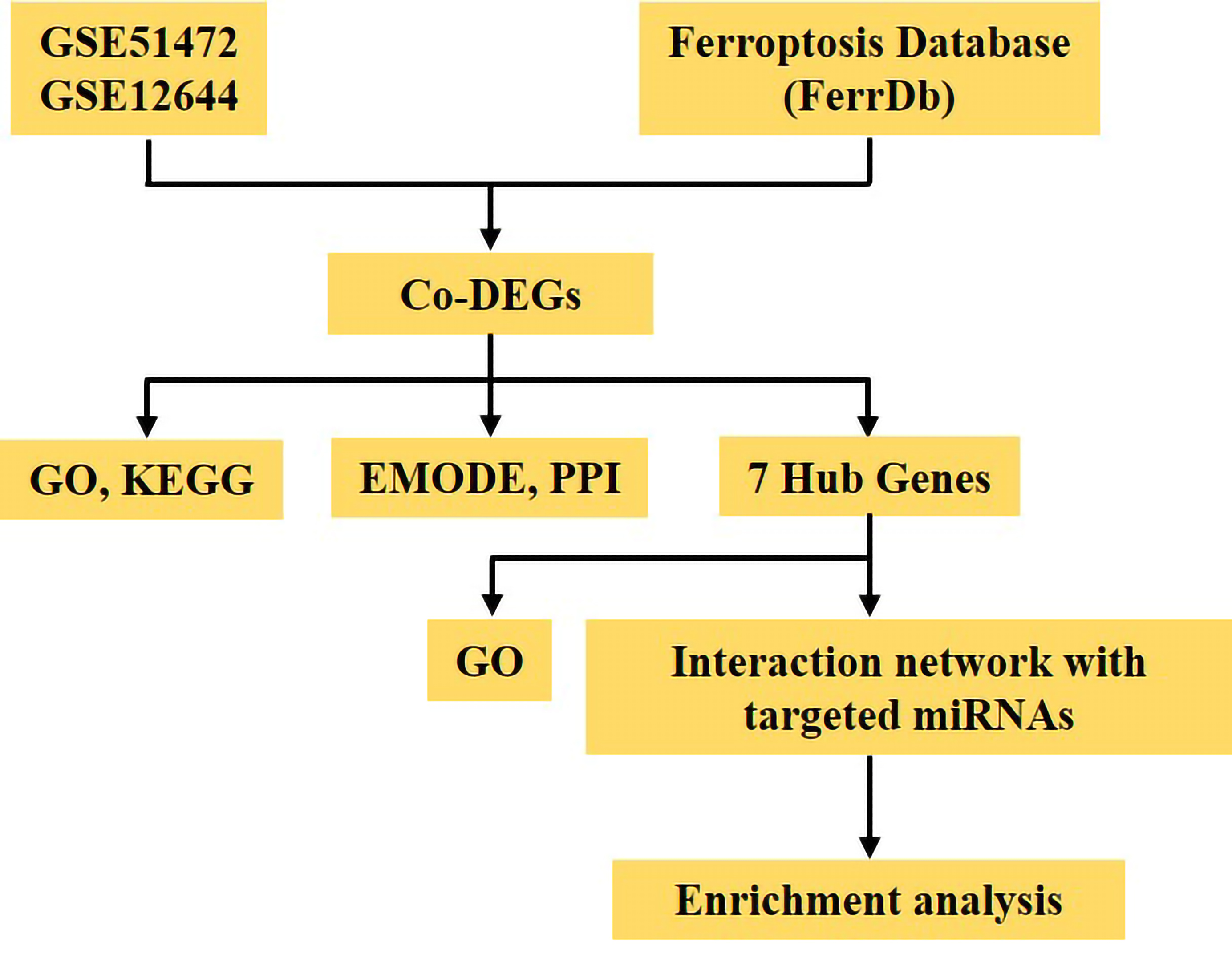

Supplement: Supplementary Figure 2 — Flowchart of data analysis. [file Image_2.TIF]
