# Supplementary material for: Potential ferroptosis key genes in calcific aortic valve disease
Source: Front Cardiovasc Med. 2022 Aug 8;9:916841. doi: 10.3389/fcvm.2022.916841 (PMC9395208; doi:10.3389/fcvm.2022.916841)
Supplement: Supplementary Table 1 — Specific primers of the ferroptosis DEGs used for qRT-PCR. [file Table_1.DOCX]

**Supplemental TABLE 1 |** Specific primers of ferroptosis DEGs used for qRT-PCR.

| **Gene name** | **Primer Sequence** | |
| --- | --- | --- |
| HMOX1 | Forward Primer | AGTTCAAGCAGCTCTACCGC |
|  | Reverse Primer | GCAACTCCTCAAAGAGCTGGAT |
| RRM2 | Forward Primer | ACTATGCTCTCCCTCCGTGT |
|  | Reverse Primer | GCTGCTTTAGTTTTCGGCTCC |
| SLC2A3 | Forward Primer | ATCCTTCCTGAGGACGTGGAG |
|  | Reverse Primer | TATCAGAGCTGGGGTGACCTTC |
| DPP4 | Forward Primer | GGAATGCCAGGAGGAAGGAATC |
|  | Reverse Primer | GCAGACCAGGACCGGAACA |
| IL-6 | Forward Primer | ACTCACCTCTTCAGAACGAATTG |
|  | Reverse Primer | CCATCTTTGGAAGGTTCAGGTTG |
| ENPP2 | Forward Primer | GAGAGCAGAAGGATGGGAGGAAG |
|  | Reverse Primer | GTCATGGCAGCAACTGGTATAGC |
| ALOX5 | Forward Primer | CGGCACTGACGACTACATCT |
|  | Reverse Primer | TATGAATCCACCGCGCCAC |
| CAPG | Forward Primer | GATAGGCCAGCAGTCATCCC |
|  | Reverse Primer | TCCACACCACCTTCCTGGTA |
| BID | Forward Primer | AAGGAGGAAGCGGGTAGTCG |
|  | Reverse Primer | ACCGTTGTTGACCTCACAGT |
| CYBB | Forward Primer | GAGTTGTCATCACGCTGTGC |
|  | Reverse Primer | GCCCACGTACAATTCGTTCAG |
| NCF2 | Forward Primer | GGTGCCCCTTTCAGAAGACA |
|  | Reverse Primer | GAAAGCCTTGGTCACCCACT |
| HIF-1α | Forward Primer | AGAGGTTGAGGGACGGAGAT |
|  | Reverse Primer | GACGTTCAGAACTTATCCTACCAT |
| NQO1 | Forward Primer | TGGTTTGGAGTCCCTGCCAT |
|  | Reverse Primer | CACTGCCTTCTTACTCCGGAAGG |
| AKR1C1 | Forward Primer | AGCCAGCACACTAAGCCTATCTAC |
|  | Reverse Primer | AGTTCTTCATCCTCCAGCCATCC |
| PSAT1 | Forward Primer | TCCTTGTACAACACGCCTCC |
|  | Reverse Primer | ATGCCTCCCACAGACACGTA |
| PLIN4 | Forward Primer | CAACCTTCGGAAAAGATGGTGTC |
|  | Reverse Primer | CGTAAGTGCAGACCGAGTGG |
| CDO1 | Forward Primer | TCTCTGCGACGACATCCTTACG |
|  | Reverse Primer | AGCACTTCGGTCTGTTCCATCTC |
| VLDLR | Forward Primer | AGCAGTATCAGAGGTCAGTGTTCC |
|  | Reverse Primer | CCAATTCCGCCACATCAAGTAGC |
| PRKAA2 | Forward Primer | TCCTGTTCTGCTGCTGGCTTAC |
|  | Reverse Primer | GGCGAGGTGAAACTGAAGACAATG |
| TF | Forward Primer | TGAGATGGTGTGCAGTGTCG |
|  | Reverse Primer | CGCTTCGTTTGCCGCAATG |
| ANGPTL7 | Forward Primer | GCGTGTAGAGATGGAGGACTG |
|  | Reverse Primer | TTCCCCAGGAAGAGGCGATA |
| GAPDH | Forward Primer | ACAACTTTGGTATCGTGGAAGG |
|  | Reverse Primer | GCCATCACGCCACAGTTTC |
